# Direct curvature measurement of the compartments in bamboo-shaped multi-walled carbon nanotubes via scanning probe microscopy

**DOI:** 10.1038/s41598-020-79692-2

**Published:** 2021-01-12

**Authors:** Jae-Won Jang

**Affiliations:** Division of Physics and Semiconductor Science, Dongguk University, Seoul, 04620 Republic of Korea

**Keywords:** Characterization and analytical techniques, Atomic force microscopy, Surfaces, interfaces and thin films

## Abstract

Bamboo-shaped multi-walled carbon nanotubes (BS-MWCNTs) have compartmented structures inherently obtained during their catalytic growth, and the curvature of the compartmented structure is known to be determined by the morphology of the metal catalysts. In this study, the inside curvature of the BS-MWCNTs was directly measured through scanning probe microscopy (SPM). The surface of the compartment structures of BS-MWCNTs has discontinuous graphene layers and different frictional force levels depending on the curvature direction. That of the inside curvature can be directly observed through tribological analysis by adding and subtracting the lateral force microscopy images obtained on opposite sides along the axial direction of the BS-MWCNT (diameter of 500 nm). This tells us the direction of the inside curvature of the BS-MWCNT, which was also confirmed by identifying the growth direction of the BS-MWCNTs via scanning electron microscopy. Our demonstration implies that SPM can give the same insight into the structural characterization of nanomaterials that is relatively inexpensive and more user-friendly than currently used methods.

## Introduction

Due to the catalytic growth with a nitrogen supply during carbon nanotube (CNT) synthesis^1–7^, bamboo-shaped multi-walled CNTs (BS-MWCNTs), which have a compartmented structure inside the tube similar to that in bamboo, have been reported^8–16^. Their unique structure and properties, such as a relatively large surface area and defect sites, have drawn increasing attention and led to their utilization in many applications, including their use as an absorbent for organic pollutants^13,17^, for hydrogen storage^10,18^, in lithium-ion battery electrodes^12^, and in sensors^11^. In detail, BS-MWCNTs have many defect sites near the partition part of the compartments because the multi-layered graphene sheets comprising the partition part are open-ended at the surface of the BS-MWCNTs^1,8,9,15^, which inevitably develops to maintain the curvature of the compartmented part. These defect sites appear regularly owing to the periodicity of the compartmented structure in BS-MWCNTs^1,2,8–10,15^. In addition, it is known that the curvature of the compartmented part is determined by the curvature of the catalyst; graphene sheets are synthesized on catalytic metal nanoparticles and peel off as the growth process of the BS-MWCNTs proceeds^1^. Typically, the curvature of the compartment part protrudes if the catalytic metal nanoparticles are located at the bottom of the BS-MWCNTs^1^. Observation of this structural uniqueness of the BS-MWCNTs is currently only possible via high-resolution transmission electron microscopy (HR-TEM).

Herein, it is reported that the inside curvature of the compartment in the BS-MWCNTs can be directly measured through scanning probe microscopy (SPM). This technique has been widely used to characterize the mechanical properties of nanomaterials^19–30^. Especially, it has been reported that structural information underneath the surface of the nanomaterial can be observed through atomic force microscopy (AFM)^15,21,23,24^ as well as scanning tunneling microscopy (STM)^19,20,26^. In addition, the frictional force mode for AFM can be directly used to observe the structural characters of nanomaterials^22,25,27–29,31,32^. According to previous reports on the HR-TEM characterization of BS-MWCNTs^1,2,8–10,15^, the defect sites composed of open-ended graphene sheets are connected with the compartmented structure. It is anticipated that this open-ended graphene sheet region on the surface of the BS-MWCNTs has different frictional force levels depending on the scanning direction when the tip scans along the tube axis of the BS-MWCNT. Based upon this scanning direction-dependent frictional force, it has been demonstrated that the direction of the inside curvature of a BS-MWCNT can be directly observed through the tribological analysis of lateral force microscopy (LFM) images. Moreover, since it is known that the inside curvature structure of BS-MWCNTs is dependent on their growing direction^1,33^, the direction of the inside curvature can be verified by the growth direction of the BS-MWCNT measured via scanning electron microscopy (SEM). Our demonstration means that the structural characterization of nanomaterials can be conducted with a relatively inexpensive and user-friendly tool such as AFM rather than HR-TEM.

## Results and discussion

### The principles of LFM imaging

**Figure 1:**
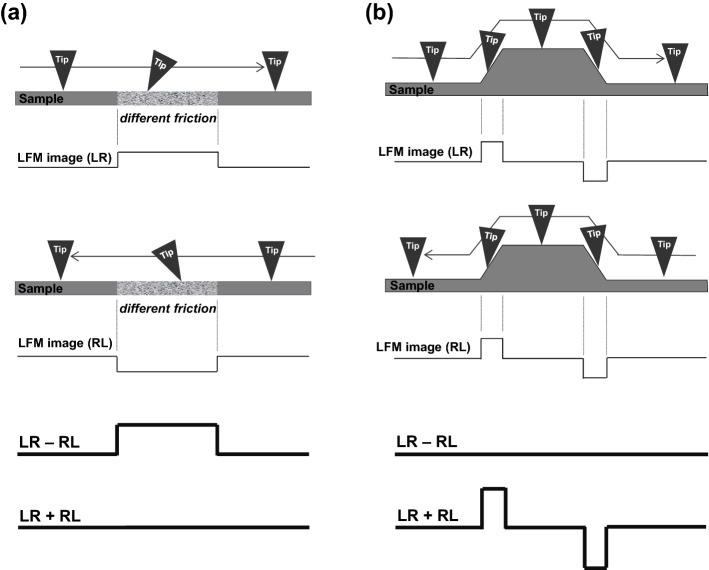
Schematics showing the principles of lateral force microscopy (LFM) imaging. The intensity of LFM during the imaging is brought about by the tip (**a**) moving over different regions of friction and (**b**) moving up from and down to different levels. LR, left-to-right; RL, right-to-left. LR − RL and LR + RL are the resulting images by subtracting and adding the LFM images, respectively.

In theory, the intensity of the LFM is acquired from the slant of the tip during the scanning process (Fig. 1). Two possible situations can cause the tip to slant: when the tip moves to a different frictional region on the sample surface (Fig. 1a) and when it moves up (or down) to a different level on the sample surface (Fig. 1b)^34,35^. Figure 1a presents the situation where the tip moves to a relatively high frictional region. Because the tip is dragged by the high frictional region on the sample surface, it slants toward the direction it is moving in regardless of it being forward (from left-to-right; LR) or backward (from right-to-left; RL). In addition, the tip slanting due to the high friction is reflected as a positive or a negative value in LFM images scanned either forward (LR) or backward (RL), respectively^34,35^. Hence, when the LFM image of LR is subtracted by that of RL, the resulting LFM image (LR − RL) has a more positive value for a relatively high frictional region in the sample whereas when they are added together, the resulting LFM image (LR + RL) has the same value in both frictional regions, as shown in Fig. 1a. However, in the other situation of the tip slanting by moving up to and down from a different level (Fig. 1b), the tip drags when it is moved up, resulting in a positive or a negative value in the LFM image depending on its scan direction^34,35^. Conversely, the tip slips when it moves down, resulting in a negative or a positive value in the LFM image depending on its scan direction similar to the case of moving up. Hence, the subtracted LFM image (LR − RL) produces the same value without notifying whether the tip moved up or down whereas the added LFM image (LR + RL) produces more positive and negative values when the tip moves up or down regions related to its scan direction, as shown in Fig. 1b. In short, the ascent, descent, and different frictional regions of the sample surface can be discriminated by subtracting or adding the LFM images obtained from the forward and backward scans.

### Tribological analysis of the compartmented structure of the BS-MWCNTs

**Figure 2:**
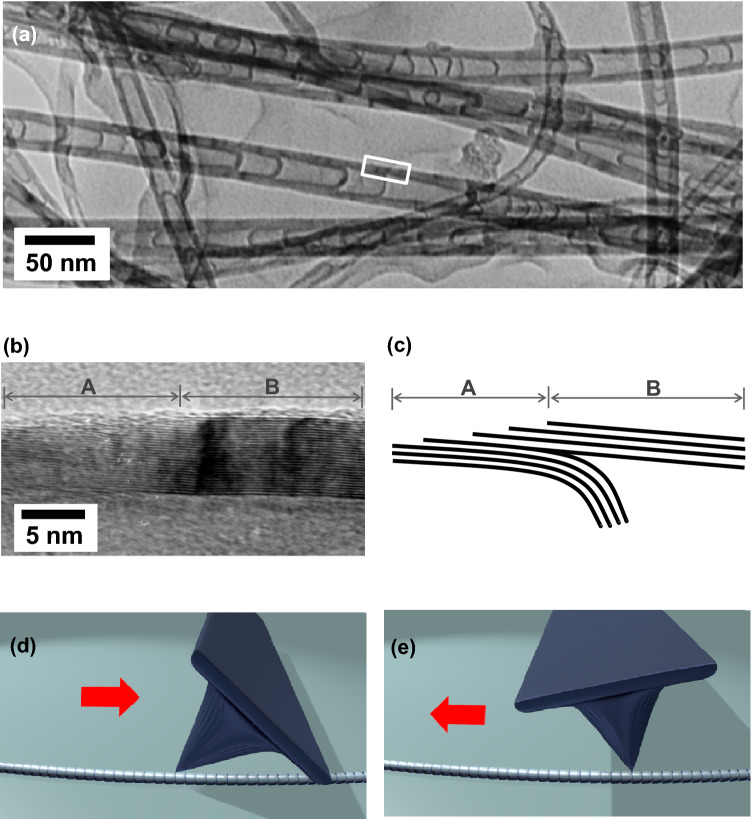
(**a**) A transmission electron microscopy (TEM) image of bamboo-shaped multi-walled carbon nanotubes (BS-MWCNTs) and (**b**) a zoomed-in high-resolution TEM image of the white-box area in (**a**). Schematic representations of (**c**) the high-resolution TEM image in (**b**) and when the tip moves (**d**) from the tail to the head and (**e**) in the opposite direction of a BS-MWCNT. A and B in (**b**) and (**c**) denote discontinuous and continuous regions of the graphene sheets, respectively.

As shown in the TEM image in Fig. 2a, there are well-developed periodically compartmented structures inside of the BS-MWCNTs. In detail, the compartmented parts of the BS-MWCNTs are curved in the same direction, which is determined by the surface curvature of the catalytic metal nanoparticles while the BS-MWCNTs are growing, with balancing between the surface and vertical growths on the surface of the catalysts^1^. As carbon molecules, decomposed from gas source (i.e. C_2_H_4_) during thermal chemical vapor deposition (CVD), are diffused on the surface of the catalytic metal nanoparticles, graphene sheets are formed on the surface of the nanoparticles (the surface growth). The diffusion of carbons accelerates into the reaction zone of the catalytic particle with carbons suppling through the edge of the catalytic particles. Then, the graphene sheets can lift off the catalytic particle by accumulating stress under the graphite sheets; meanwhile, end of the graphene sheets can grow to the vertical direction with a successive carbon suppling to the edge of the catalytic particles (the vertical growth). Due to bulk diffusion of the carbons through the catalytic metal nanoparticles, graphene sheets on the surface of the nanoparticles can be formed again via the surface growth. Under a stable condition of thermal CVD, the surface and vertical growths of the graphene sheets on the catalytic metal nanoparticles are continuously processed and compartment structures can be repeatedly generated. Owing to curvature of the compartment structures is determined by the surface curvature of the catalytic metal nanoparticles, the compartmented parts always protrude to the head of the BS-MWCNTs, especially when the catalyst is located at the bottom of the BS-MWCNTs^1^. In addition, the HR-TEM image of the wall near the compartmented structure of the BS-MWCNT in Fig. 2b shows that the multi-walled graphene sheets are inclined with discontinuous and continuous regions having developed near the compartment structure, as presented schematically in Fig. 2c. The open-ended graphene sheets are sequentially stacked in a herringbone structure, showing that the discontinuous and continuous regions were regularly formed in the BS-MWCNTs.

Owing to the herringbone-like stack of open-ended graphene sheets in the discontinuous region of the surface of the BS-MWCNTs, asymmetrical friction would exist when the AFM tip moves up and down along the axial direction of the BS-MWCNT. Supposedly, the friction when the tip moves up to the protruding direction of the compartmented structure (from the tail to the head of the BS-MWCNT) is relatively larger than that when it moves down away from the protruding direction (from the head to the tail of the BS-MWCNT), as shown schematically in Fig. 2d, e. The tip would be asymmetrically tilted due to the asymmetrical friction during back-and-forth scanning along the axial direction of the BS-MWNCT.

### Principles of LFM imaging of the compartmented structure of the BS-MWCNTs

**Figure 3:**
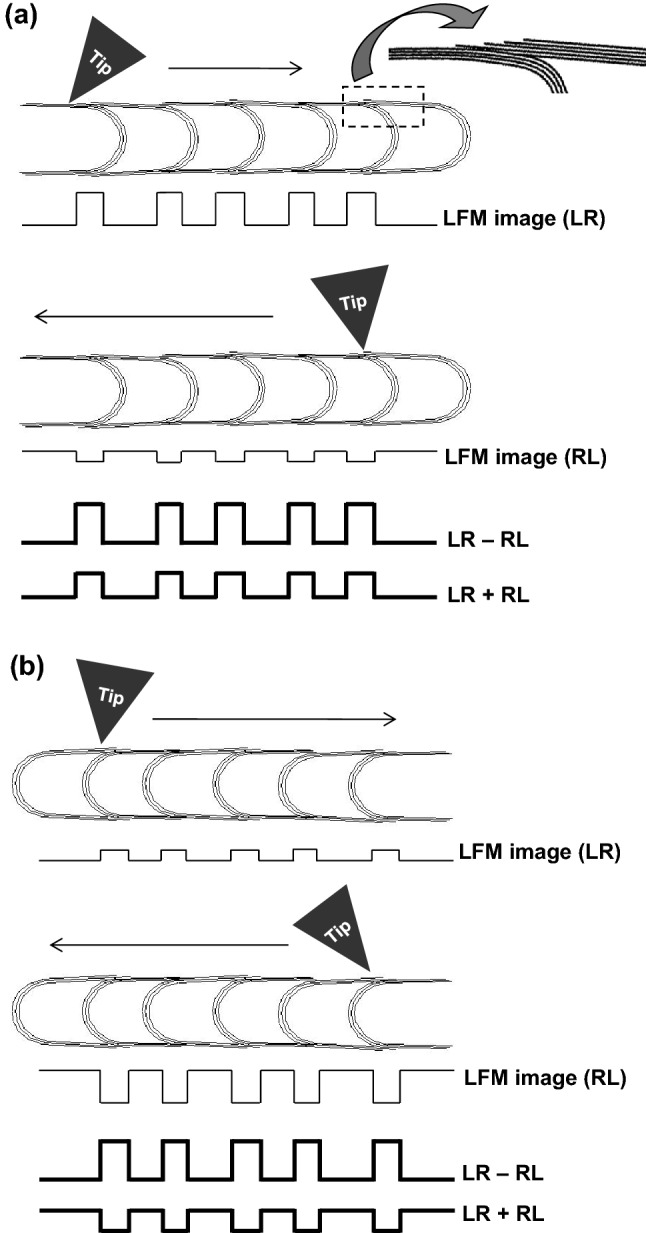
A schematic illustrating the principles of lateral force microscopy (LFM) imaging carried out by scanning along the axial direction of the bamboo-shaped multi-walled carbon nanotube (BS-MWCNT) when the protruding direction of the compartmented structure is (**a**) to the right and (**b**) to the left. LR, left-to-right; RL, right-to-left. LR − RL and LR + RL are the resulting images by subtracting and adding the LFM images, respectively.

The intensity of the LFM image of the BS-MWCNT can be predicted and the curvature of the compartment structure (the growing direction) of the BS-MWCNT characterized by considering the asymmetrical friction dependent on the scan direction at the surface of the BS-MWCNTs, as represented in Fig. 3. When the protruding direction of the compartment structure of the BS-MWCNT is on the right (Fig. 3a), the intensity of the LFM image in the discontinuous region composed of the open-ended graphene sheets near the compartmented structure of the BS-MWCNT is larger when the tip scans toward the head of the BS-MWCNT (LR) than that when the tip scans to the tail of the BS-MWCNT (RL) due to the asymmetrical friction denoted in Fig. 2b–e. If the LFM images are subtracted from each other (LR − RL), then positive values will be obtained for the discontinuous regions near the compartmented structures. Also, if the LFM images are added each other (LR + RL), positive values with relatively smaller than that of LR − RL will be obtained for the discontinuous regions near the compartmented structures as described in Fig. 3a. Correspondingly, the opposite scenario occurs when the protruding direction of the compartment structure of the BS-MWCNT is on the left (Fig. 3b); the intensity of the LFM image in the discontinuous region is positive and smaller when the tip scans toward the head of the BS-MWCNT (LR) than when it scans toward the tail (RL). Hence, the intensity of the added LFM image (LR + RL) in the discontinuous region is negative while the subtracted LFM images (LR − RL) is positive and relatively large, as described in Fig. 3b. Consequently, the curvature in the protruding direction of the compartmented structure of the BS-MWCNT can be directly observed by monitoring the subtracted and added LFM images (LR − RL, LR + RL) when back-and-forth scanning is carried out along the axial direction of the BS-MWCNT.

### Characterization of the curvature of the compartmented structure of the BS-MWCNTs

**Figure 4:**
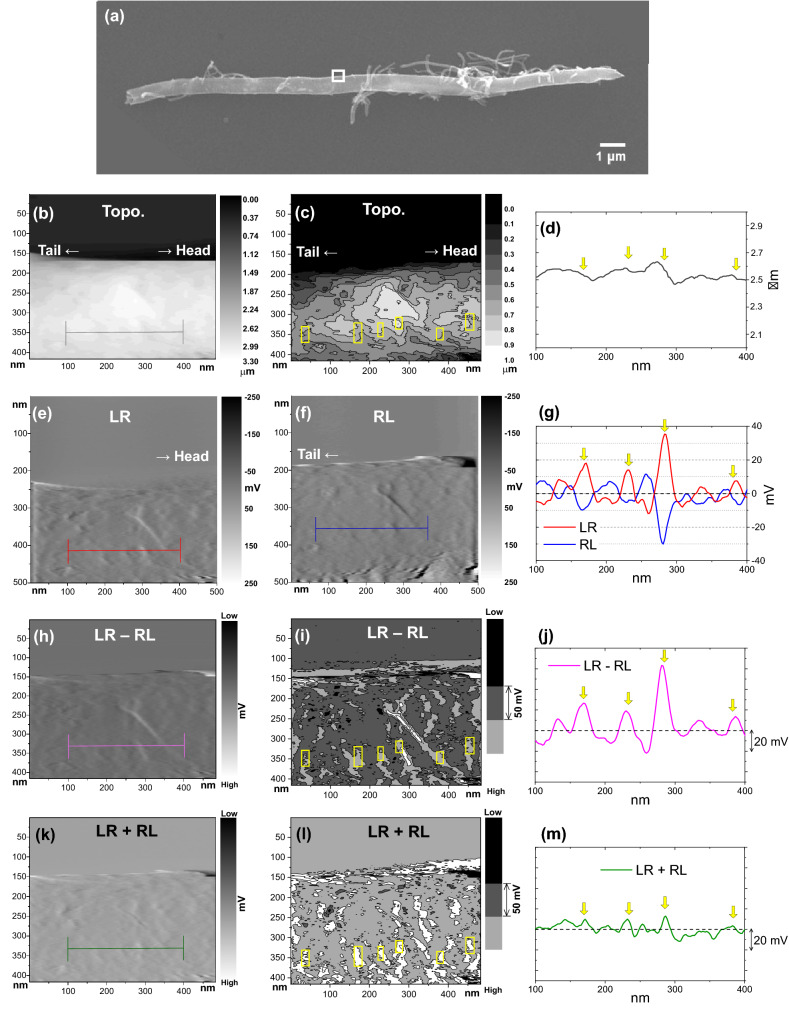
(**a**) A scanning electron microscopy (SEM) image of a bamboo-shaped multi-walled carbon nanotube (BS-MWCNT) on highly oriented pyrolytic graphite (HOPG). (**b**) An atomic force microscopy (AFM) topographic image (Topo.). (**c**) A respective contour plot of (**b**). (**d**) A line profile obtained from the dark grey line in (**a**). (**e**–**f**) LFM images measured with scanning from (**e**) left-to-right (LR) and (**f**) right-to-left (RL). (**g**) Line profiles obtained from the red and blue lines in (**g**–**f**). The x-axis of the line profile graph is matched with (**g**). (**h**) A subtracted LFM image (LR − RL). (**i**) A respective contour plot of (**h**). (**j**) A line profile obtained from the magenta line in (**h**). (**k**) An added LFM image (LR + RL). (**l**) A respective contour plot of (**k**). (**m**) A line profile obtained from the olive line in (**k**). Measurements for Topo., LR, RL, LR − RL, and LR + RL are carried out on the white-box area in (**a**). The directions to the tail and head of the BS-MWCNT are denoted with arrows. The groups of the yellow boxes in (**c**, **i**, **l**) have been placed at the same positions. The yellow arrows in (**d**, **g**, **j**, **m**) indicate the same positions. The black dashed line in (**g**, **j**, **m**) indicates zero level.

AFM and SEM measurements of a single BS-MWCNT were carried out to demonstrate direct observation on the curvature of the compartmented structure, as shown in Fig. 4. Figure 4a exhibits an SEM image of the BS-MWCNT carried out to examine the growing direction of the BS-MWCNT. It can be seen that the head and tail of the BS-MWCNT are located on the right and left in Fig. 4a, respectively, which means that the growing direction of the BS-MWCNT was from left-to-right as is the protruding direction of the compartmented structure. To characterize the curvature of the compartmented structures, topological AFM in conjunction with back-and-forth LFM scanning was carried out on the white-box region in Fig. 4a; the AFM topographic image (Topo.), the LFM images with scanning from left-to-right (LR) and from right-to-left (RL), the subtracted LFM image (LR − RL), and the added LFM image (LR + RL) are presented in Fig. 4b, e, f, h, k, respectively. For a clearer perspective, the images are re-displayed with contour plots in Fig. 4c, i, l and line profiles obtained from Topo., LR, RL, LR − RL, and LR + RL are displayed in Fig. 4d, g, j, m, respectively. The substrate as well as the BS-MWCNT were specifically scanned to exactly match the background offset of the back-and-forth scanned LFM images (LR and RL). Furthermore, the girth region of the BS-MWCNT is not flat, which could result in visual artifacts during the imaging. Hence, we considered the intensity of the subtracted and added LFM images in the regions away from the girth of the BS-MWCNT. However, the line profile (Fig. 4d) obtained from the dark-grey line in Fig. 4b shows that the surface of the BS-MWCNT is not perfectly flat. The yellow arrows shown in the line profiles are used as markers to denote the same positions. In addition, the back-and-forth scanned LFM images (LR and RL) are displayed in Fig. 4e, f, respectively. The LFM images of LR and RL have an offset to the vertical and horizontal directions. The line profiles, obtained at the same position of the BS-MWCNT (the red line in Fig. 4e and the blue line in Fig. 4f), are simultaneously represented in Fig. 4g. The line profile of LR is mainly positive, and that of RL is mainly negative, where magnitude of the negative value of the line profile of RL is relatively small compared to the positive value of the line profile of LR at the same position (the yellow arrows). The observed tendency of the line profile of LR and RL matches with the scheme of line profiles of LFM imaging shown in Fig. 3a; that is the curvature direction of BS-MWCNT is to the right. However, baselines of the line profiles of LR and RL are not zero; which is not anticipated in Fig. 3. For the scheme of line profiles of LFM imaging of BS-MWCNT shown in Fig. 3, assumptions that the surface of BS-MWCNT is perfectly flat and asymmetrical friction expected at the discontinuous graphene sheets near the compartment structures only influences on the line profile are considered. In fact, the surface of the BS-MWCNT is not perfectly flat as shown in Fig. 4d, which could be a cause of the non-zero baseline of the line profile of LR and RL (Fig. 4g). As well as the yellow arrows in the line profiles, the yellow boxes in Fig. 4c,i, l denote the same regions in Topo., LR − RL, and LR + RL images (the four yellow boxes in the middle of the images are overlapped with the yellow arrows in the line profiles). As shown in Fig. 4j, m, the line profile of LR − RL represents positive value and that of the LR + RL represents relatively smaller positive value as indicated by the yellow arrows, which matches the LFM image described in Fig. 3a. According to the scheme of line profiles of LFM imaging of BS-MWCNT (Fig. 3), the line profile of LR − RL will be same and positive regardless of the curvature direction of BS-MWCNT. Moreover, as described in Fig. 1, the subtracted LFM image (LR − RL) is always larger than zero; if different friction of the surface is dominant then the LR − RL is positive (Fig. 1a), and the LR − RL is zero if different level of the surface is dominant (Fig. 1b). Hence, it can be assumed that negative value of the LR − RL represents an experimental error; for examples, negative LR − RL can be obtained when the line profiles of LR and RL is not equal (the line profile of RL is larger than that of LR that may have occurred from different surface frictions or levels). Inequivalent LR and RL could result from several factors such as misalign between tip and BS-MWCNT, asymmetric tip geometry, and etc. Even if the negative LR − RL can be regarded as an experimental error, Fig. 4j represents that magnitude of the positive LR − RL values is relatively larger than that of the negative LR − RL values. Therefore, the characterization of curvature direction of the BS-MWCNT through the subtracted and added LFM images could be regarded as valid, and observations of the curvature of the compartmented structure via LFM imaging are the same as those via SEM imaging, with the protruding direction of the compartment structure being to the right. In addition, the distances between the yellow boxes are 143, 64, 43, 100, and 71 nm from the left, respectively, which are similar values to the previously reported compartmented distances of the BS-MWCNTs fabricated under the same synthetic conditions (Fig. S2)^9^.

## Conclusions

In conclusion, we reported that the periodic discontinuous region on the surface of BS-MWCNTs originating from the compartmented structure has asymmetrical friction depending on the scan direction (back-and-forth along the tube axis direction). From a tribological perspective, the curvature of the compartmented (protruding) direction can be confirmed by directly observing whether the intensity of the subtracted and added back-and-forth scanned LFM images is positive or negative. The asymmetrical friction depending on the scan direction related to terminating status of the graphene sheets can be a good example that SPM is a useful tool to investigate tribology in molecular scale. Moreover, these results suggest that SPM (including the AFM family) can be used as a convenient and relatively inexpensive tool for the structural characterization of nanomaterials compared to electron microscopy.

## Methods

### Sample preparation

As the first step for the synthesis of BS-MWCNTs, SiO_2_/Ti multi-film (300 nm/1 mm) was deposited on pieces of Si wafer (p-type (100)). Afterward, the Fe catalyst was deposited on the SiO_2_/Ti deposited Si substrates, and the BS-MWCNTs were then synthesized on the Fe catalyst at 950 °C by using thermal CVD under acetylene (C_2_H_2_) gas with a flow rate of 30 sccm for 10 min just after ammonia (NH_3_) pretreatment under normal pressure^16^. Before AFM measurements, the BS-MWNTs were sonicated in acetone for 1 h and drop-casted onto highly oriented pyrolytic graphite (HOPG). The outmost layer of the HOPG was exfoliated with scotch tape to clean it just before the drop-casting of the BS-MWCNTs.

### Structural characterization

Contact mode AFM (SPA400, Seiko Instruments Inc.) equipped with a pyramidal shaped tip (PNP-TR, Nanoworld AG, Switzerland: the tip radius of curvature is blow 10 nm) was carried out to capture LFM images of the BS-MWCNTs. A tip cantilever approached to the surface of the BS-MWCNT while crossing the axes of the tip cantilever and the BS-MWCNT. Next, LFM and topographic AFM images of an individual BS-MWCNT were measured at the same time while scanning along the axial direction of the BS-MWCNT on the clean HOPG. The LFM and AFM images were alternately measured twice as the scanning proceeded from left-to-right and vice versa.

SEM (Hitachi S-4300) was carried out to determine the growing direction of the BS-MWNT to ascertain the positions of the head and/or tail. TEM (JEOL 200 CX) and HR-TEM (Philips CM20T, 200 kV) were performed to observe the compartmented structures in the BS-MWCNTs and the open-ended graphene sheets nearby.

## Supplementary Information

Supplementary information.

## Data Availability

The datasets used and/or analysed during the current study are available from the corresponding author on reasonable request.
