# Supplementary material for: Direct curvature measurement of the compartments in bamboo-shaped multi-walled carbon nanotubes via scanning probe microscopy
Source: Sci Rep. 2021 Jan 12;11:701. doi: 10.1038/s41598-020-79692-2 (PMC7804926; doi:10.1038/s41598-020-79692-2)
Supplement: Supplementary file 1 — Supplementary information. [file 41598_2020_79692_MOESM1_ESM.pdf]

Supplementary Data

Direct Curvature Measurement of the Compartments in Bamboo-Shaped Multi-Walled Carbon Nanotubes via Scanning Probe Microscopy

Jae-Won Jang*

Division of Physics and Semiconductor Science, Dongguk University, Seoul 04620, Republic of Korea.

*Corresponding author: Jae-Won Jang

Tel: +82-2-2260-3220

E-mail: jwjang@dgu.ac.kr ; jwjang@dongguk.edu

I. Scanning electron microscopy (SEM) images of a bamboo-shaped multi-walled carbon nanotube (BS-MWCNT)

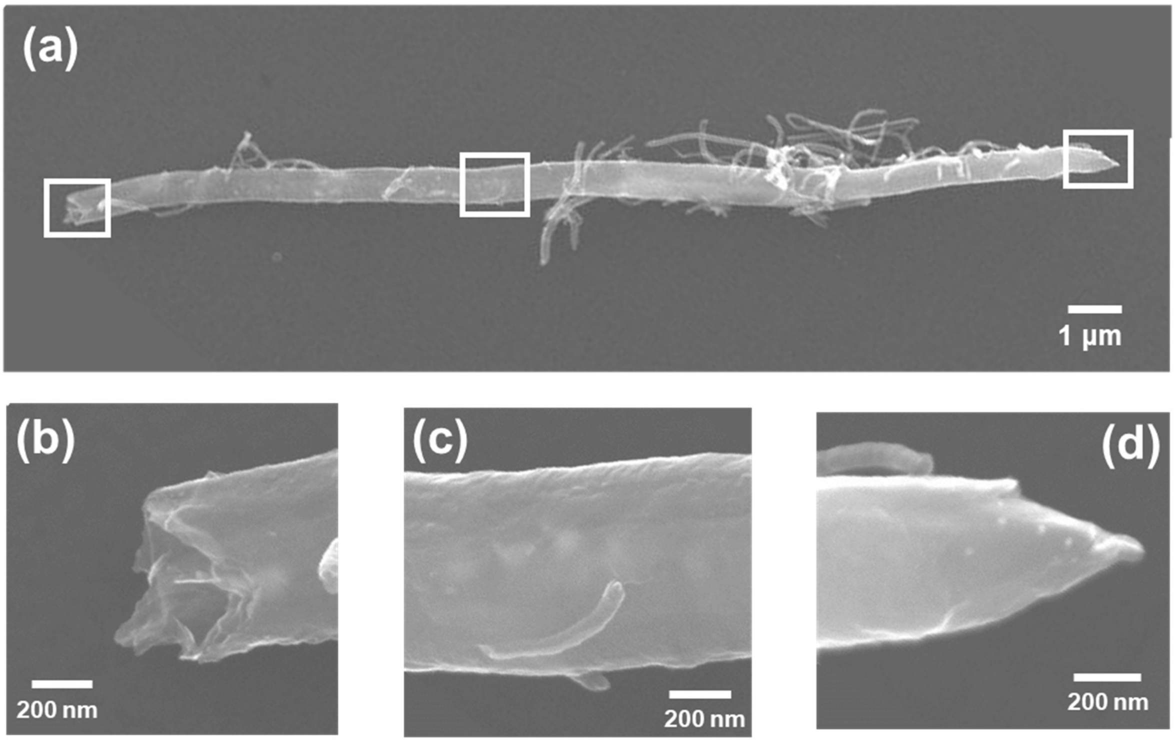

Figure S1. (a) Zoomed scanning electron microscopy (SEM) images of the bamboo-shaped multi-walled carbon nanotube (BS-MWCNT) shown in Figure 4(a) are provided to more clearly observe the (b) tail, (c) middle, and (d) head sections.

II. Diameter-dependent compartment distance of the BS-MWCNTs

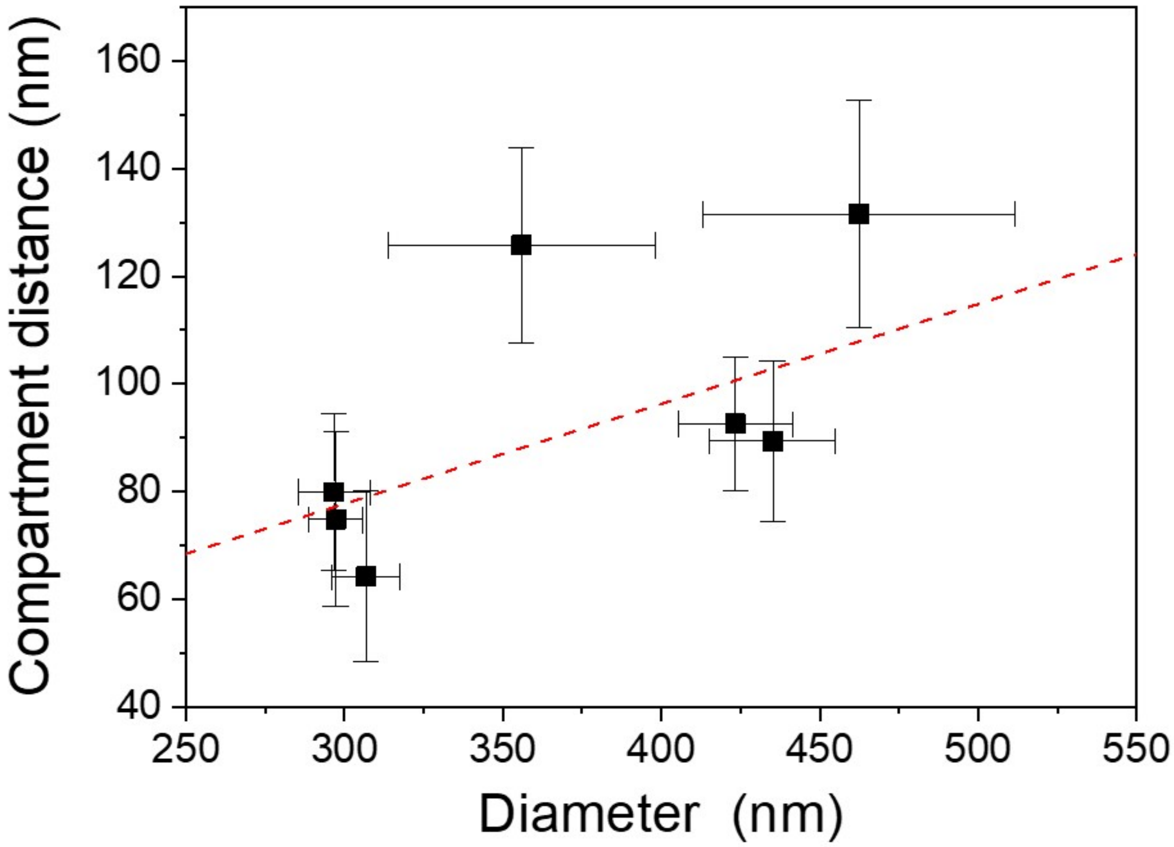

Figure S2. Diameter-dependent compartment distance of the bamboo-shaped multi-walled carbon nanotubes (BS-MWCNTs) synthesized at 950 °C via thermal chemical vapor deposition under acetylene (C_2H_2) gas at a flow rate of 30 sccm for 10 min just after ammonia (NH_3) pretreatment under normal pressure [Jang J W, *et. al.* 2003 Solid State Commun. 127 29-32]. The red dash-line is a linearly fitted line.
